# Comment on “Prevalence and Cognitive Bases of Subjective Memory Complaints in Older Adults: Evidence from a Community Sample”

**DOI:** 10.1155/2014/240215

**Published:** 2014-08-13

**Authors:** Ashima Nehra, Sakshi Chopra, Harsimarpreet Kaur

**Affiliations:** Neurosciences Centre, All India Institute of Medical Sciences, Room No. NS-718, Ansari Nagar, New Delhi 110029, India

It was a pleasure reading your paper entitled* “Prevalence and Cognitive Bases of Subjective Memory Complaints in Older Adults: Evidence from a Community Sample,”* which was published on 27 April 2014. It was a very detailed paper about the incidence of subjective memory complaints (SMCs) in older adults and how importantly SMCs could have a prognostic value in predicting objective memory decline and dementia [1, 2].

It was very interesting to note the process of making subjects as a part of the study, who are drawn from memory clinics, biased in finding a higher prevalence of SMC as subjects are more likely to have memory impairments, whereas, on the other hand, how population based studies may be biased in finding a lower prevalence of SMC's as the subjects are more willing to participate in research and are also healthier than the patient groups (subjects drawn from memory clinics).

However, in the methodology, the clear inclusion and exclusion criteria were not mentioned for the subjects who were made a part of the study. There was no mention about any comorbid conditions or disorders as well, which the participants who were included in the study had, as this information can be important for further having any correlations between the existing comorbid conditions/disorders and SMCs. Also, the subjects were divided into two groups, younger adults and older adults, but there was no mention on what criteria this was based. According to the World Health Organization, any person who is 60 years old and above can be called an elderly or an older population [3], so we would like to know why subjects who were 60 years old and above were not included and why 65 years old and above subjects were enrolled.

Moreover, in the methods, there were 3 hypotheses of the study, but there was no mention about them being accepted or rejected. The third objective which stated that gender and objective memory would be associated with a greater risk for SMCs in women than in men was rejected after obtaining the results. The same could have been stated in the results and conclusions.

The paper was also very comprehensive about the data and results, but nothing was mentioned about the subjects who actually performed low in the objective neuropsychological evaluation, who turned out to have dementia, whether they referred to a neurology clinic after that for any treatment/intervention or not. The future directions and implications of the study could have been highlighted and how the study can be benefitted to the general population to make substantial generalizations would further strengthen the research.

Overall, it was a very interesting study, and we hope that these can be done in India as well, soon.
